# Adipsic Hypernatremia With Neurologic Manifestations in a Patient With Severe COVID-19 Pneumonia

**DOI:** 10.7759/cureus.21484

**Published:** 2022-01-21

**Authors:** Mohammad M Kasim, Gustav F Strandvic, Ismail Y Mahmood, Muayad K Ahmad, Abdulqadir J Nashwan

**Affiliations:** 1 Medicine, Hamad Medical Corporation, Doha, QAT; 2 Nursing, Hamad Medical Corporation, Doha, QAT

**Keywords:** pneumonia, encephalopathy, sars-cov2, covid-19, adipsic hypernatremia

## Abstract

This report describes a case of a 50-year-old man with hypertension who was admitted with a history of fever, chills, and shortness of breath and tested positive for COVID-19. Shortly after resolving his acute respiratory distress syndrome (ARDS), he developed adipsic hypernatremia with associated confusion, lethargy, and weakness. COVID-19 is a serious disease that mainly targets the respiratory system; however, we must not overlook its effects on other organ systems. When the etiology of hypernatremia is unclear, it requires extensive workup and monitoring, and the lack of rapid correction can cause serious and irreversible neurological damage.

## Introduction

At the end of 2019, a novel coronavirus (SARS‐CoV‐2) broke out of Wuhan city in China. Rapidly spreading throughout the world, COVID-19 was declared a pandemic in March 2020 by the World Health Organization (WHO) [1,2]. It is no doubt that COVID-19 can present in an array of neurological manifestations, and those who are critically ill are most vulnerable to developing such complications [3]. We report a case of adipsic hypernatremia with neurological manifestations in a patient with severe COVID-19 pneumonia, which was challenging to diagnose and treat.

## Case presentation

A 50-year-old man with a known history of hypertension on calcium channel blockers presented to the emergency department with three days history of fever, non-productive cough, generalized weakness, and loss of appetite. On examination, he was tachycardic and tachypneic with oxygen saturation of 87% on room air and 99% on 5 liters simple face mask. There was bilateral coarse crepitation bilaterally on chest auscultation. General examination was unremarkable. Over the next 24-hours post-presentation, his oxygen requirements and work of breathing increased (respiratory rate of 50 cycles/min and saturation of 92% on 15 liters mask with reservoir), which required endotracheal intubation and mechanical ventilation. A nasopharyngeal polymerase chain reaction swab was positive for SARS-CoV-2. The diagnosis of COVID-19 acute respiratory distress syndrome was made, and the patient was admitted to the intensive care unit with a PaO2/FiO2 ratio of 150. After recovery from his respiratory illness and successful extubation, the patient developed adipsic hypernatremia up to 164 mmol/L (reference range 135-145 mmol/L) that was not caused by iatrogenic fluid administration. It was associated with confusion, lethargy, and generalized weakness.

Investigations

**Figure 1:**
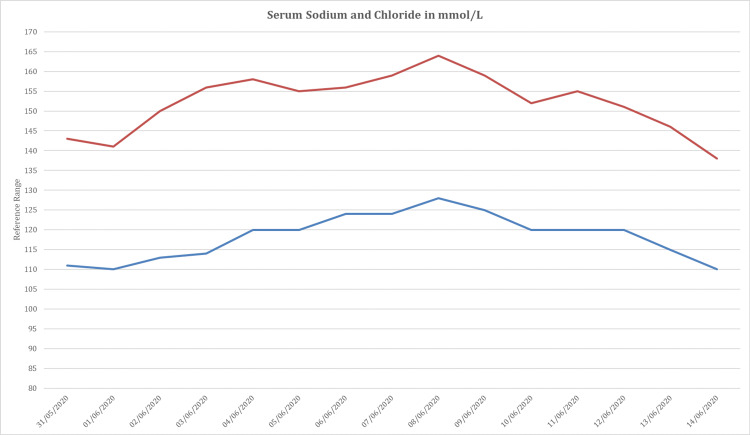
Daily serum sodium and chloride concentrations (mmol/L) post recovery from SARS-COV-2 associated acute respiratory distress syndrome (ARDS). Serum sodium continued to climb after extubating the patient and peaked at 164 mmol/L despite adequate hydration.

Post orotracheal extubation, the patient was confused with serum sodium above 155 mmol/L (reference range 135-145 mmol/L). He was adipsic, and his sodium remained high despite receiving parenteral hypotonic fluids and free water orally (Figure 1).

**Figure 2:**
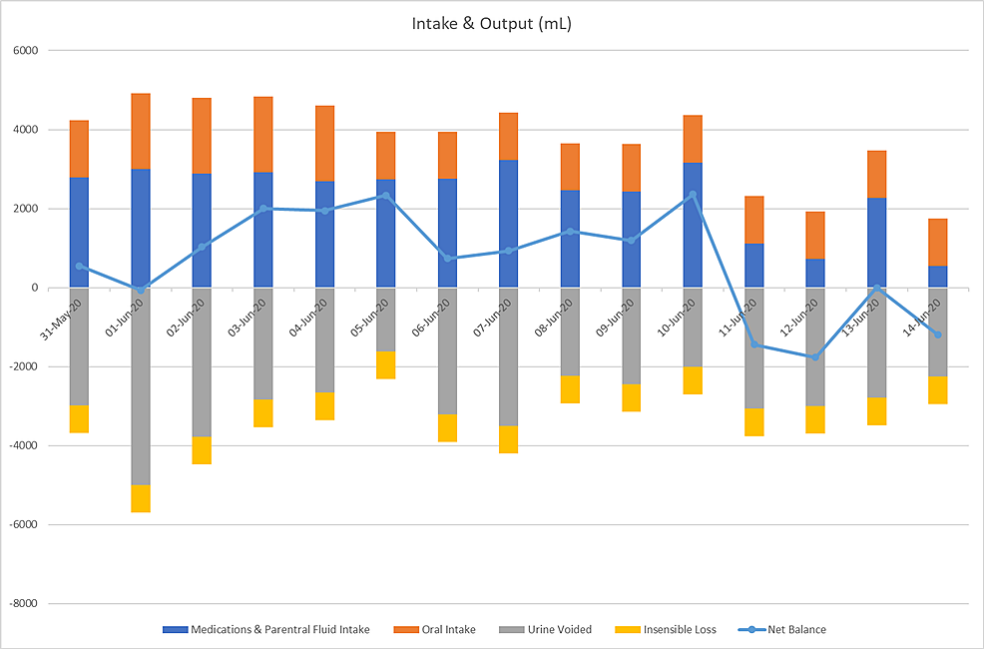
Daily intake and output in milliliters. The patient received parenteral intravenous fluid in the form of dextrose 5% water; the estimated insensible daily loss was 700 mL/day.

His net fluid balance was positive with normal urine output (Figure 2). 

**Figure 3:**
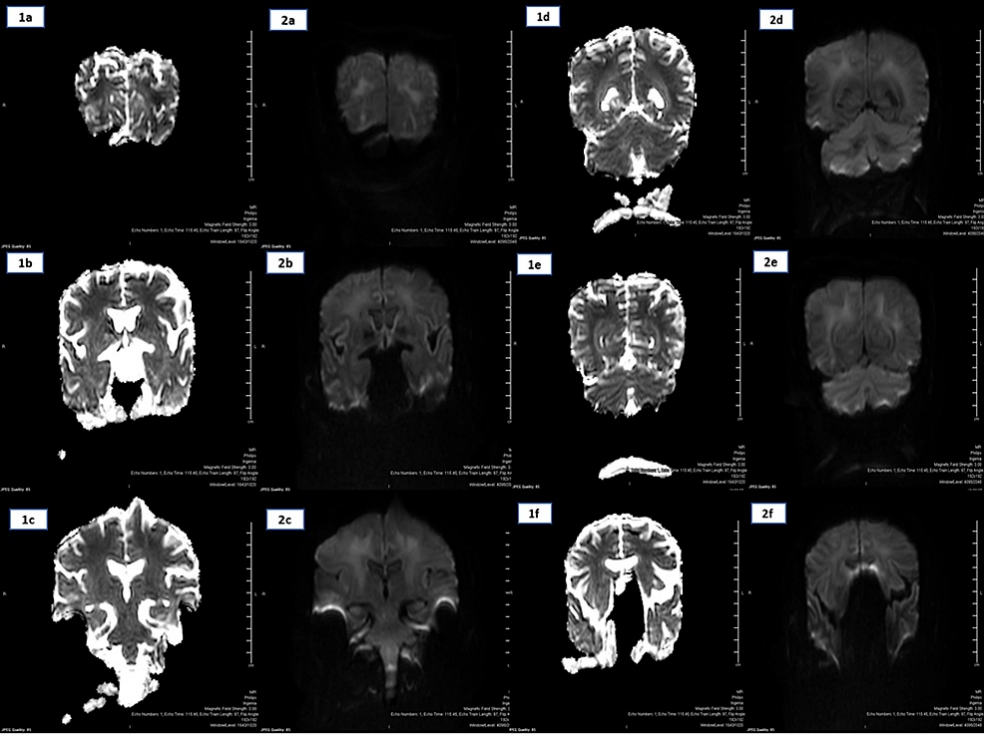
(1a-1f): Apparent diffusion coefficient (ADC) map arrow showing bilateral symmetrical supratentorial diffusion restriction in the centrum semiovale and occipital subcortical regions. (2a-2f) Coronal MRI diffusion-weighted imaging showing bilateral symmetrical supratentorial diffusion restriction in the centrum semiovale and occipital subcortical regions.

A 24-hour urine collection results were normal (measured serum osmolality: 350 mOsm/kg, calculated serum osmolarity: 346 mOsm/kg, urine osmolality: 520 mOsm/kg, 24-hour urine volume: 2321 mL, 24-hour urine creatinine: 13.20 mmol/24 hrs, 24-hour urine sodium: 139 mmol/24 hrs). In addition, adrenocorticotropic, serum cortisone, and thyroid hormone levels were normal. A CT of the head ruled out an acute cerebrovascular insult that could account for his confusion. An MRI ruled out a hypothalamic lesion causing adipsic hypernatremia and showed patterns of restrictive diffusion associated with encephalopathy (Figure 3).

Differential diagnosis

**Figure 4:**
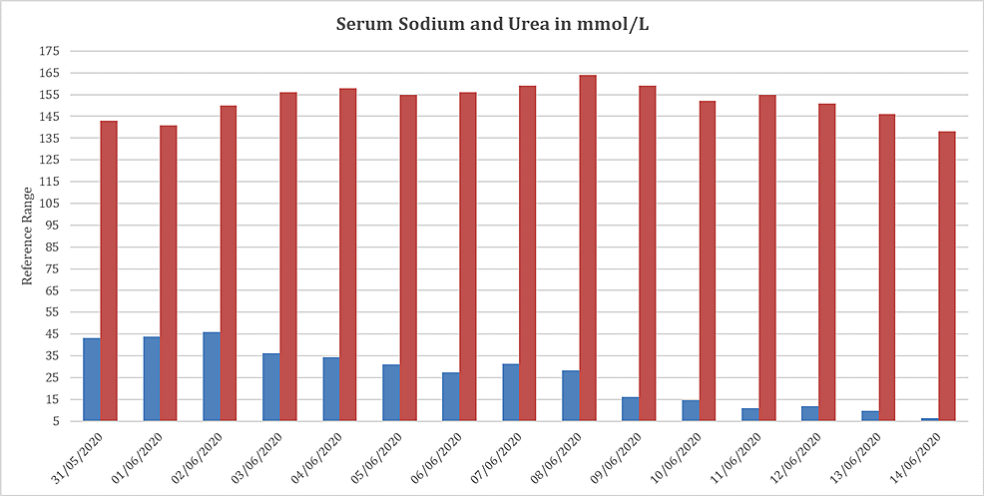
Daily serum sodium and urea concentrations (mmol/L) post recovery from SARS-COV-2 acute respiratory distress syndrome (ARDS). Serum sodium continued to climb after extubating the patient and peaked at 164 mmol/L despite adequate hydration.

In most cases, hypernatremia is caused by unreplaced water losses; however, the patient did not have a gastrointestinal or significant insensible loss. Hypernatremia can also be caused by iatrogenic hypertonic sodium solutions or oral salt intake; however, the patient was on hypotonic fluid replacement and free water orally; thus, iatrogenic sodium overload was not a factor in his persistent hypernatremia (total fluids received: 89,978, total output: 78,314, net balance: 11,664) (Figure 2). Serum glucose measurements were normal, and the patient did not receive mannitol to cause osmotic diuresis. Despite a mild to moderate elevation in serum urea concentration, which was resolving, it did not explain the patient's lack of thrusting the hypernatremia that was resistant to fluid replacement (Figure 4).

The fact that the patient had a normal urine output with normal urine osmolality and 24-hour urine sodium concentration made the diagnosis of central or nephrogenic diabetes insipidus very unlikely, and with that regard, patients with central or nephrogenic diabetes insipidus typically have a normal thrust mechanism. Since the patient had an impaired thrust mechanism, we suspected a central cause of his hypernatremia; however, CT and MRI of the head ruled out a hypothalamic lesion or a structural abnormality that would cause primary hypodipsia or osmoreceptor dysfunction. Following the MRI, we initially thought that the patient's encephalopathy might be caused by either direct viral invasion or metabolic-related causes. However, with the lack of other clinical signs, the slow but steady improvement the patient was showing in response to fluid replacement, and the fact that two consecutive sets of polymerase chain reaction samples taken from the nasopharynx were negative for SARS-CoV-2 post respiratory recovery for the infection, all hindered us from tapping his cerebrospinal fluid unnecessarily. Furthermore, a defect of thrust usually occurs in populations with congenital acquired hypothalamic lesions which often dictates forced water intake to maintain normal sodium concentration. A careful neurologic and radiologic evaluation was performed to rule out a central cause.

Outcome and follow up

The patient was sedated with propofol, midazolam, and fentanyl during the early period of mechanical ventilation and dexmedetomidine was used during the weaning from the ventilator. The use of cisatracurium during the first day was necessary to control the high ventilatory requirements and peak pressure. He received a seven-day course of ceftriaxone and azithromycin, methylprednisolone, and intravenous immunoglobulin for five days (the remainder of medications used were routine medications used for ICU care like chemical DVT prophylaxis, bowel regime, and a proton pump inhibitor). The patient was mechanically ventilated for 10 days and the length of stay in a critical care setting was 22 days. After extubation, he was on an oral diet and free water, he had to be persuaded by the nurses to drink water due to lack of thirst. The patient had received free water through a nasogastric tube with his enteral feed during the intubation period. The patient was discharged from rehabilitation with normal neurological and functional capacity. Two months after discharge; the patient was completely asymptomatic; He can perform his daily activities and is awaiting the lifting of restrictions to resume his work as a blacksmith.

## Discussion

SARS-CoV-2 respiratory manifestations are well documented; however, there is increasing evidence that this highly infectious virus involves other systems (e.g., gastrointestinal, renal, and hepatic). Despite the limited data, multiple case reports and reviews suggest neurological involvement with an estimated incidence of 37% [4]. These findings include but are not limited to headaches, confusion, anosmia, weakness, encephalitis, seizures, encephalopathy, and cerebrovascular insults and are more common among severe cases of COVID-19 pneumonia. While severe cases are associated with excessive inflammatory responses leading to multi-organ dysfunction, we highlight this case report's neurologic and metabolic involvement.

The diverse neurological complications might be a consequence of cardiorespiratory failure, metabolic abnormalities, autoimmune response, or direct neuro-invasion by the virus. And it appears that neurologic disorders were more common in the critically ill [5]. Encephalitis is uncommonly reported, but a Japanese man with SARS-COV-2 encephalitis has been confirmed by a polymerase chain reaction in the cerebrospinal fluid [6]. Although encephalopathy is more commonly associated with an acute illness accompanied by metabolic abnormalities mostly due to a cytokine storm than with viral invasion of the brain, rare cases of the latter appear to occur.

A case series of 12 patients, confirmed to have SARS-COV-2 by polymerase chain reaction, reported the development of therapy-resistant hypernatremia (plasma sodium > 150 mmol/L) without correlation between plasma sodium levels and sodium input [7]. Therefore, it is hypothesized that the SARS-COV-2 virus binds to the angiotensin-converting enzyme 2 (ACE2) receptors in the kidneys resulting in unopposed angiotensin II accumulation and resorption of sodium in the proximal convoluted tubule [8-10].

## Conclusions

We believe that in this case, neurological manifestations were a complication of hypernatremia rather than by direct neuro-invasion since the MRI findings are common in metabolic dysfunction. The presumption is that the patient was COVID-19 negative at the time, which would have resulted in a negative cerebrospinal fluid result. Nonetheless, the presence of adipsic hypernatremia and resulting neurological manifestation were both challenging to diagnose and manage.
